# The Synthesis of Planar Four-Bar Linkage for Mixed Motion and Function Generation

**DOI:** 10.3390/s21103504

**Published:** 2021-05-18

**Authors:** Bin Wang, Xianchen Du, Jianzhong Ding, Yang Dong, Chunjie Wang, Xueao Liu

**Affiliations:** School of Mechanical Engineering and Automation, Beihang University, Beijing 100191, China; wangbin2014@buaa.edu.cn (B.W.); duxianchen@buaa.edu.cn (X.D.); jianzhongd@buaa.edu.cn (J.D.); dongyang@buaa.edu.cn (Y.D.); wangcj@buaa.edu.cn (C.W.)

**Keywords:** robotic mechanism design, linkage synthesis, motion generation, function generation

## Abstract

The synthesis of four-bar linkage has been extensively researched, but for a long time, the problem of motion generation, path generation, and function generation have been studied separately, and their integration has not drawn much attention. This paper presents a numerical synthesis procedure for four-bar linkage that combines motion generation and function generation. The procedure is divided into two categories which are named as dependent combination and independent combination. Five feasible cases for dependent combination and two feasible cases for independent combination are analyzed. For each of feasible combinations, fully constrained vector loop equations of four-bar linkage are formulated in a complex plane. We present numerical examples to illustrate the synthesis procedure and determine the defect-free four-bar linkages.

## 1. Introduction

Linkage synthesis is to determine link dimensions of the linkage that achieves prescribed task positions [1,2,3,4]. Traditionally, linkage synthesis is divided into three types [5,6], motion generation, function generation, and path generation. Each of the synthesis types has been extensively researched and has many applications in various engineering fields, but, in some situations, a hybrid task needs to be performed. A hybrid task synthesis is to design the mechanisms that accomplish two or three of linkage synthesis types simultaneously, during a single movement. Many problems in engineering practice require some combination of these problem types. For example, the pick-and-place system requires a part not only to accomplish the picking task but also to avoid obstructions during a single movement [7], which is the problem of combination of motion and path generation for four-bar linkage. In automotive fields, stowing automotive seats frequently requires a combination of motion and function generation. The hybrid task synthesis can also be applied to aeronautic and astronautic fields and so on. In this paper, we present a numerical synthesis procedure for four-bar linkage that combines motion and function generation; this method can be extended and applied to six-bar and eight-bar linkages.

To the best of the author’s knowledge, the idea of hybrid task synthesis was proposed by Smaili and Diab [8] in 2006; they divided a crank cycle into several segments, each of the segments performed one of the synthesis types. In 2013, Tong and Murray [9] presented the synthesis for combination of motion generation and path generation, which differs from Ref. [8], the combination are satisfied in one segment. Based on the foundation, Brake et al. [10] studied the Alt–Burmester problems with all possible combinations, the Alt–Burmester problem is the combination of motion generation and path generation. In 2018, Zimmerman [11] presented a graphical method that tried to synthesize four-bar linkage to satisfy any combination of these three synthesis types. However, in the above literature, the combination of motion generation and function generation for four-bar linkage was not studied completely.

Generally, there are three linkage synthesis methods which are graphical method, optimization method and numerical analytical method. The graphical method is to draw the linkage step by step under geometric constraints by the target positions on the linkage through the poles and rotation angles. Beyer [12] shows a graphical method for two coupler points and specified input and output angle changes corresponding to those two points. Zimmerman [11] used Pole and rotation angle constraints to draw the four-bar linkage in a CAD software. The graphical method is intuitive, but the steps become extremely complex when the number of task positions increases. The optimization method is to obtain an optimum linkage by building an optimization model [8,13,14,15]. For linkage synthesis, only one linkage solution is obtained usually by an optimization method.

The numerical analytical method is to formulate the kinematic constraint loop equations and solve for the appropriate link lengths and pivot locations. The research for numerical analytical method of linkage belongs to the area, numerical algebraic geometry, which was proposed by Sommese and Wampler [16] in 1996. The foundation of numerical algebraic geometry is the solving of systems of polynomial equations based on the homotopy continuation method and to obtain all isolated solutions of the polynomial systems. Liu and McCarthy [4,17] applied a numerical analytical method to solve the problem of motion generation, and the number of task positions ranges from 2 to 5. Wampler et al. [18] used an analytical method to construct the constraint equation and solved it by a homotopy method for the nine point path synthesis problem for four-bar linkages, and they proved that the complete solution of the system are obtained. Plecnik and McCarthy [19,20] presented a numerical analytical method to synthesize function generation for six-bar linkages and solved it by the polynomial homotopy solver Bertini [21].

In this paper, we present a numerical synthesis procedure for four-bar linkage that combines motion generation and function generation, which was not addressed before. The procedure is divided into two categories which are named as dependent combination and independent combination. Five feasible cases for dependent combination and two feasible cases for independent combination are analyzed. For each of feasible combinations, fully constrained vector loop equations of four-bar linkage are formulated in complex planes. In addition, we give numerical examples to illustrate the numerical procedure and determine the defect-free linkages. In what follows, we present how to perform the numerical procedure.

### 1.1. Isotropic Coordinates

**Figure 1:**
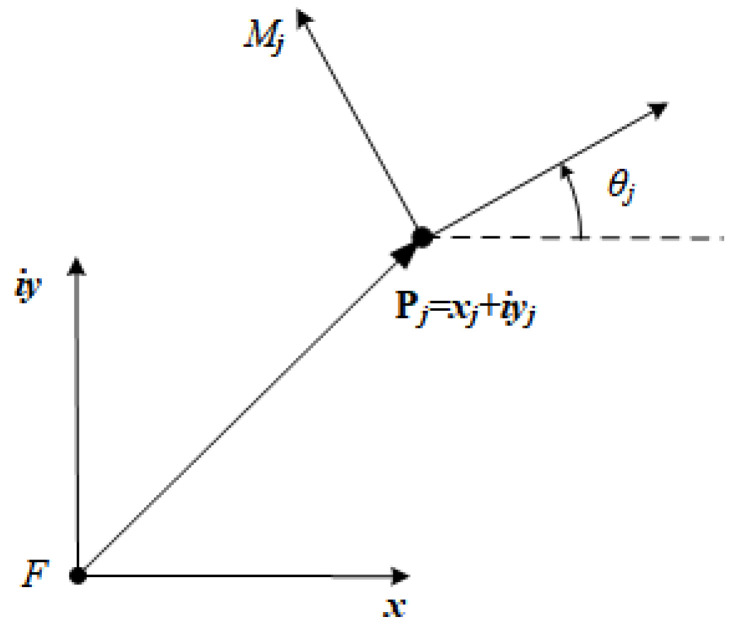
Representation form of vector and rotation operation in a complex plane.

It is convenient to use vectors in complex plane to formulate constraint equations in planar kinematics [22]. Instead of denoting a vector $P_{j}$ as $P_{j}=\left\{x_{j},y_{j}\right\}$ in a Cartesian coordinates system, we represent it as $P_{j}=x_{j}+iy_{j}$ and its conjugate ${\bar{P}}_{j}=x_{j}-iy_{j}$ in complex plane, where $i=\sqrt{-1}$, see Figure 1. Note that the length of vector $P_{j}$ can be calculated by $\sqrt{P_{j}{\bar{P}}_{j}}$. In addition, the unit vector $e^{i\theta_{j}}$ is the rotation operation that defines the rotation from the fixed frame *F* to the reference frame $M_{j}$. Applying the Euler identity, we have

(1) $$e^{i\theta_{j}}=\cos\theta_{j}+i\sin\theta_{j}.$$

The conjugate of Equation (1) denotes the rotation from the reference frame $M_{j}$ to the fixed frame *F*, which is

(2) $$e^{-i\theta_{j}}=\cos\theta_{j}-i\sin\theta_{j}.$$

### 1.2. Motion Generation

The task of motion generation is to guide the coupler link of a four-bar linkage through prescribed points and orientations. In this paper, we define the prescribed point and orientation as motion task position (MTP) which is denoted as $\left\{P_{j},\theta_{j}\right\}$, where $\theta_{j}$ is relative to the *x*-axis of the fixed frame *F*.

**Figure 2:**
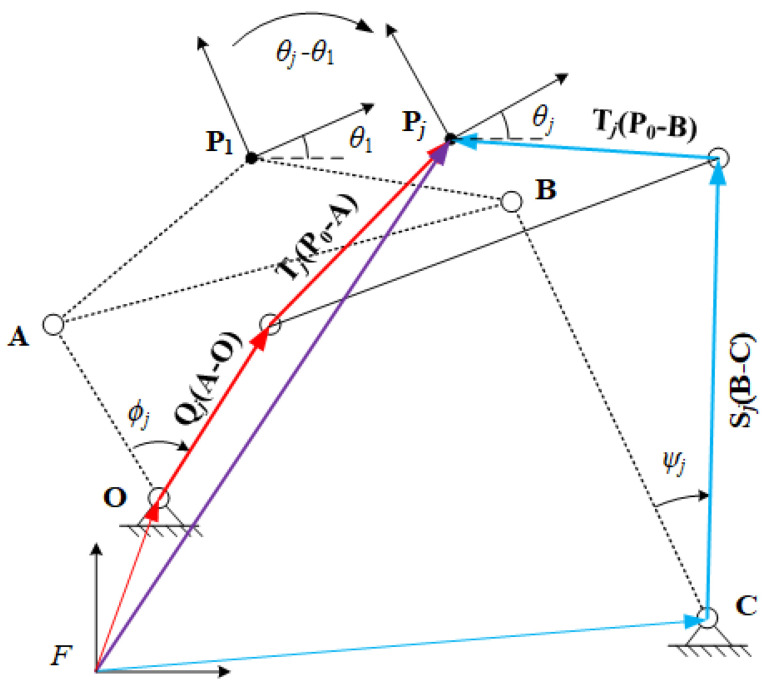
Vector diagram of a four-bar linkage at MTP $\{P_{j},\theta_{j}$}.

Figure 2 displays a four-bar linkage whose coupler link ${\mathrm{ABP}}_{1}$ is passing through MTP $\left\{P_{j},\theta_{j}\right\}$ from MTP $\left\{P_{1},\theta_{1}\right\}$. During the movement, input link OA rotates the angle of $\phi_{j}$, output link BC rotates the angle of $\psi_{j}$ and coupler link ${\mathrm{ABP}}_{1}$ rotates the angle of $\theta_{j}-\theta_{1}$, which are relative to the position $\left\{P_{1},\theta_{1}\right\}$. We define $Q_{j},S_{j}$ and $T_{j}$ as rotation operations, so

(3) $$Q_{j}=e^{i\phi_{j}},\;S_{j}=e^{i\psi_{j}}\;\mathrm{and}\;T_{j}=e^{i(\theta_{j}-\theta_{1})}.$$

The conjugates of Equation (3) denote reverse rotation with the same amount,

(4) $${\bar{Q}}_{j}=e^{-i\phi_{j}},\;{\bar{S}}_{j}=e^{-i\psi_{j}}\;\mathrm{and}\;{\bar{T}}_{j}=e^{i(\theta_{1}-\theta_{j})}.$$

At MTP $\left\{P_{j},\theta_{j}\right\}$, the constraint vector loop equations of the four-bar linkage can be formulated by

(5) $$\begin{array}{ccc} & & O+Q_{j}\left(A-O\right)+T_{j}\left(P_{1}-A\right)-P_{j}=0,\\ & & C+S_{j}\left(B-C\right)+T_{j}\left(P_{1}-B\right)-P_{j}=0. \end{array}$$

The conjugate of Equation (5) are

(6) $$\begin{array}{ccc} & & \bar{O}+{\bar{Q}}_{j}\left(\bar{A}-\bar{O}\right)+{\bar{T}}_{j}\left({\bar{P}}_{1}-\bar{A}\right)-{\bar{P}}_{j}=0,\\ & & \bar{C}+{\bar{S}}_{j}\left(\bar{B}-\bar{C}\right)+{\bar{T}}_{j}\left({\bar{P}}_{1}-\bar{B}\right)-{\bar{P}}_{j}=0. \end{array}$$

Note that $Q_{j}$ and $S_{j}$ are unit vectors, so they have the unit magnitude,

(7) $$\begin{array}{ccc} & & Q_{j}{\bar{Q}}_{j}=1,\\ & & S_{j}{\bar{S}}_{j}=1. \end{array}$$

$Q_{j}$ and $S_{j}$ can be expressed by solving Equation (5), and ${\bar{Q}}_{j}$ and ${\bar{S}}_{j}$ by Equation (6). Then, substituting these expressions into Equation (7) to eliminated $Q_{j}$, $S_{j}$ and ${\bar{Q}}_{j}$, ${\bar{S}}_{j}$,

(8) $$\begin{array}{ccc} & & \left(P_{j}-O-T_{j}\left(P_{1}-A\right)\right)\left({\bar{P}}_{j}-\bar{O}-{\bar{T}}_{j}\left({\bar{P}}_{1}-\bar{A}\right)\right)-\left(A-O\right)\left(\bar{A}-\bar{O}\right)=0,\\ & & \left(P_{j}-C-T_{j}\left(P_{1}-B\right)\right)\left({\bar{P}}_{j}-\bar{C}-{\bar{T}}_{j}\left({\bar{P}}_{1}-\bar{B}\right)\right)-\left(B-C\right)\left(\bar{B}-\bar{C}\right)=0. \end{array}$$

where j=2,…,m, *m* denotes the maximum number of MTPs that a four-bar linkage can achieve during a movement. In Equation (8), $T_{j}$, $P_{j}$, ${\bar{T}}_{j}$ and ${\bar{P}}_{j}$ are specified by the designer, so there are eight unknowns, O, A, B, C, $\bar{O}$, $\bar{A}$, $\bar{B}$ and $\bar{C}$. Note that O and $\bar{O}$ are two independent unknowns for solving the constraint loop equations, so are A, B, and C. When m=5, the number of equations is equal to the number of unknowns, which means the loop equations are fully constrained. This indicates a four-bar linkage can achieve at most five MTPs.

### 1.3. Function Generation

The task of function generation of four-bar linkage is to coordinate the rotation angles of input link and output link. In this paper, we define the position where the four-bar linkage is at and the linkage’s input angle is $\phi_{k}$ and output angle $\psi_{k}$ as function task position (FTP). FTP is denoted as $\left\{\phi_{k},\psi_{k}\right\}$, where the angles are relative to the *x*-axis of the fixed frame *F*.

**Figure 3:**
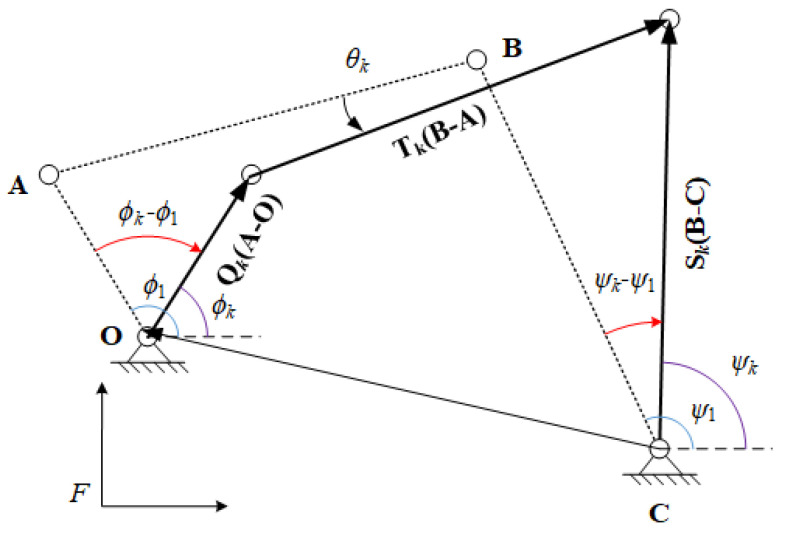
Vector diagram of a four-bar linkage at FTP $\{\phi_{k},\psi_{k}$}.

Figure 3 displays a four-bar linkage is at FTP $\left\{\phi_{k},\psi_{k}\right\}$ from FTP $\left\{\phi_{1},\psi_{1}\right\}$. During the movement, the input link rotates the angle of $\phi_{k}-\phi_{1}$, the output links rotates the angle of $\psi_{k}-\psi_{1}$, and the coupler link rotates the angle of $\theta_{k}$, which are relative to the FTP $\left\{\phi_{1},\psi_{1}\right\}$. Thus, the corresponding rotations can be denoted as

(9) $$\begin{array}{ccc} & & Q_{k}=e^{i(\phi_{k}-\phi_{1})},\;S_{k}=e^{i(\psi_{k}-\psi_{1})},\;T_{k}=e^{i\theta_{k}},\\ & & {\bar{Q}}_{k}=e^{-i(\phi_{k}-\phi_{1})},\;{\bar{S}}_{k}=e^{-i(\psi_{k}-\psi_{1})},\;{\bar{T}}_{k}=e^{-i\theta_{j}}. \end{array}$$

The constraint loop equation for function generation can be formulated by

(10) $$\begin{array}{c} O-C+Q_{k}\left(A-O\right)+T_{k}\left(B-A\right)-S_{k}\left(B-C\right)=0. \end{array}$$

The conjugate of Equation (10) is

(11) $$\begin{array}{c} \bar{O}-\bar{C}+{\bar{Q}}_{k}\left(\bar{A}-\bar{O}\right)+{\bar{T}}_{k}\left(\bar{B}-\bar{A}\right)-{\bar{S}}_{k}\left(\bar{B}-\bar{C}\right)=0. \end{array}$$

The unit vector $T_{j}$ has the unit magnitude,

(12) $$\begin{array}{c} T_{k}{\bar{T}}_{k}=1. \end{array}$$

T and ${\bar{T}}_{k}$ can be expressed by respective solving Equations (10) and (11), then substituting the expressions into Equation (12) to eliminate T and ${\bar{T}}_{k}$,

(13) $$\begin{array}{c} \left(S_{k}\left(B-C\right)-O+C-Q_{k}\left(A-O\right)\right)\left({\bar{S}}_{k}\left(\bar{B}-\bar{C}\right)-\bar{O}+\bar{C}-{\bar{Q}}_{k}\left(\bar{A}-\bar{O}\right)\right)\\ -\left(B-A\right)\left(\bar{B}-\bar{A}\right)=0. \end{array}$$

where k=2,…,n, *n* represents the maximum number of FTPs that a four-bar linkage can achieve. Note that the fixed pivots of the four-bar linkage must be specified in advance to measure the angles of input and output for function generation. Thus, in Equation (13), O, C, $\bar{O}$, $\bar{C}$, $Q_{k}$, $S_{k}$, ${\bar{Q}}_{k}$ and ${\bar{S}}_{k}$ are specified by the designer. The unknowns are A, B, $\bar{A}$ and $\bar{B}$. When n=5, the loop equations are fully constrained; therefore, a four-bar linkage can achieve at most five FTPs.

## 2. The Synthesis of Mixed Generation

In Section 2, motion generation and function generation are synthesized separately, and a four-bar linkage can achieve at most five MTPs or five FTPs. In this section, we explore the relationship of combination between the number of MTPs and FTPs. Here, the mixed synthesis process is divided into two categories, dependent combination and independent combination. In what follows, we show how to synthesize and analyze these two types of mixed generation.

### 2.1. Dependent Combination

The task of mixed generation is also to determine the link dimensions of the four-bar linkage that achieves *m* MTPs and *n* FTPs during a period of movement. The dependent combination means that there is at least a common task position (CTP) that the linkage moves through a MTP and a FTP simultaneously. On the contrary, there is no CTP during the movement, which is called independent combination.

Let *t* denote the number of CTPs, and select one of CTPs as the first task position. For the rest of t−1 CTPs relative to the first task position, we have the constraints,

(14) $$\begin{array}{ccc} & & O+Q_{l}\left(A-O\right)+T_{l}\left(P_{1}-A\right)-P_{l}=0,\\ & & C+S_{l}\left(B-C\right)+T_{l}\left(P_{1}-B\right)-P_{l}=0,\\ & & \bar{O}+{\bar{Q}}_{l}\left(\bar{A}-\bar{O}\right)+{\bar{T}}_{l}\left({\bar{P}}_{1}-\bar{A}\right)-{\bar{P}}_{l}=0,\\ & & \bar{C}+{\bar{S}}_{l}\left(\bar{B}-\bar{C}\right)+{\bar{T}}_{l}\left({\bar{P}}_{1}-\bar{B}\right)-{\bar{P}}_{l}=0,\;l=1,\ldots ,t. \end{array}$$

where the unknowns O, A, B, C, $\bar{O}$, $\bar{A}$, $\bar{B}$ and $\bar{C}$ denote the vectors and their conjugates of the four-bar linkage at the selected CTP. Here, $Q_{l}=e^{i(\phi_{l}-\phi_{1})}$, $S_{l}=e^{i(\psi_{l}-\psi_{1})}$ and $T_{l}=e^{i(\theta_{l}-\theta_{1})}$. If t=1, the Equation (14) always holds because $Q_{1}=e^{0}=1$, $S_{1}=e^{0}=1$ and $T_{1}=e^{0}=1$.

According to Equation (8), the constraint equations for the rest of m−t MTPs relative to the first task position are

(15) $$\begin{array}{ccc} \left(P_{j+t}-O-T_{j}^{M}\left(P_{1}-A\right)\right)\left({\bar{P}}_{j+t}-\bar{O}-{\bar{T}}_{j}^{M}\left({\bar{P}}_{1}-\bar{A}\right)\right)-\left(A-O\right)\left(\bar{A}-\bar{O}\right)=0, & & \\ \left(P_{j+t}-C-T_{j}^{M}\left(P_{1}-B\right)\right)\left({\bar{P}}_{j+t}-\bar{C}-{\bar{T}}_{j}^{M}\left({\bar{P}}_{1}-\bar{B}\right)\right)-\left(B-C\right)\left(\bar{B}-\bar{C}\right)=0, & & \\ j=1,\ldots ,m-t. & & \end{array}$$

where $T_{j}^{M}$ denote the coupler link rotations of the rest of MTPs relative to the selected CTP.

According to Equation (13), the constraint equations for the rest of n−t FTPs relative to the first task position are

(16) $$\begin{array}{c} \left(S_{k}^{F}\left(B-C\right)-O+C-Q_{k}^{F}\left(A-O\right)\right)\left({\bar{S}}_{k}^{F}\left(\bar{B}-\bar{C}\right)-\bar{O}+\bar{C}-{\bar{Q}}_{k}^{F}\left(\bar{A}-\bar{O}\right)\right)\\ -\left(B-A\right)\left(\bar{B}-\bar{A}\right)=0,\;k=1,\ldots ,n-t. \end{array}$$

where $Q_{k}^{F}$ and $S_{k}^{F}$ denote the input rotations and output rotations of the rest of FTPs relative to the selected CTP, respectively.

Combining Equations (14)–(16), there are eight unknowns, namely O, A, B, C, $\bar{O}$, $\bar{A}$, $\bar{B}$ and $\bar{C}$. To satisfy that the mixed equations are fully constrained, the relationship between the number of unknowns and number of equations is

(17) $$\begin{array}{c} 8=4(t-1)+2(m-t)+(n-t). \end{array}$$

There are three cases for the value of *t*, which are t=1, t=2 and t=3. For case of t=1, the combination of {t=1,m=5,n=1} is equivalent to motion generation. The combinations {t=1,m=4,n=3} and {t=1,m=3,n=5} are feasible. Note that the combinations of {t=1,m=2,n=7} and {t=1,m=1,n=9} are infeasible because a four-bar linkage achieve at most FTPs, namely n≤5. For case of t=2, there are two feasible combinations which are {t=2,m=4,n=2} and {t=2,m=3,n=4}. For case of t=3, there is only a combination {t=3,m=3,n=3}. The feasible dependent combinations of MTPs and FTPs are listed in Table 1.

#### 2.1.1. The Case of t=1

When t=1, Equation (14) can be discarded. The mixed constraint equations are the combination of Equations (15) and (16) with the index {t=1,m=4,n=3} or {t=1,m=3,n=5}. Here, the mixed constraint equations are a polynomial equations system which includes eight equations and eight unknowns, and the degree of each equation is 2. According to Bezout theory [23], the upper limit of the number of the solution sets is $2^{8}=256$. In kinematic synthesis, all isolated solution sets for the constraint equations should be obtained. In addition, the solutions should be checked to find those which can be formed as a four-bar linkage to achieve prescribed MTPs and FTPs in sequence and smoothly [24,25]. The constraint equations for the cases of t=1 can be solved by polynomial homotopy method to obtain all isolated solution sets.

#### 2.1.2. The Case of t=2

When t=2, Equation (14) are linear equations, and O, $\bar{O}$, C, $\bar{C}$ can be expressed by using A, B, $\bar{A}$, $\bar{B}$, which are

(18) $$\begin{array}{ccc} & & O=\frac{P_{2}-Q_{2}A-T_{2}\left(P_{1}-A\right)}{1-Q_{2}},\\ & & \bar{O}=\frac{{\bar{P}}_{2}-{\bar{Q}}_{2}\bar{A}-{\bar{T}}_{2}\left({\bar{P}}_{1}-\bar{A}\right)}{1-{\bar{Q}}_{2}},\\ & & C=\frac{P_{2}-S_{2}B-T_{2}\left(P_{1}-B\right)}{1-S_{2}},\\ & & \bar{C}=\frac{{\bar{P}}_{2}-{\bar{S}}_{2}\bar{B}-{\bar{T}}_{2}\left({\bar{P}}_{1}-\bar{B}\right)}{1-{\bar{S}}_{2}}. \end{array}$$

Substituting Equation (18) into Equations (15) and (16) can eliminate O, $\bar{O}$, C and $\bar{C}$, which decreases the number of unknowns to four. Now, A, $\bar{A}$, B, $\bar{B}$ can be obtained by solving Equations (15) and (16). As there are only four unknowns, the equations system can be solved using a homotopy method or directly by the command Nsolve in Mathematica software. After the results of A, $\bar{A}$, B, $\bar{B}$ are obtained, O, $\bar{O}$, C, and $\bar{C}$ can be obtained easily by solving Equation (18).

#### 2.1.3. The Case of t=3

For case {t=3,m=3,n=3}, only Equation (14) are applied. As all equations are linear, the conjugate equations can be omitted. Thus, the constraint equations are

(19) $$\begin{array}{ccc} & & O+Q_{2}\left(A-O\right)+T_{2}\left(P_{1}-A\right)-P_{2}=0,\\ & & C+S_{2}\left(B-C\right)+T_{2}\left(P_{1}-B\right)-P_{2}=0,\\ & & O+Q_{3}\left(A-O\right)+T_{3}\left(P_{1}-A\right)-P_{3}=0,\\ & & C+S_{3}\left(B-C\right)+T_{3}\left(P_{1}-B\right)-P_{3}=0. \end{array}$$

It is easy to solve the equations to obtain the results of O, A, B, C because of the linear equations.

### 2.2. Independent Combination

The independence of mixed generation is a task to determine four-bar linkages that achieve *m* MTPs and *n* FTPs without any CTP during the movement, namely t=0. Here, we select one of MTPs as the first MTP $\left\{P_{1},\theta_{1}\right\}$, and one of FTPs as the first FTP $\left\{\phi_{1},\psi_{1}\right\}$. In the four-bar linkage, O, C are fixed pivots, and A, B are moving pivots. Thus, we define the moving pivots at the first MTP as $A_{M}$, $B_{M}$, and at the first FTP as $A_{F}$, $B_{F}$.

For *m* MTPs, we have

(20) $$\begin{array}{c} \left(P_{j}-O-T_{j}\left(P_{1}-A_{M}\right)\right)\left({\bar{P}}_{j}-\bar{O}-{\bar{T}}_{j}\left({\bar{P}}_{1}-{\bar{A}}_{M}\right)\right)-\left(A_{M}-O\right)\left({\bar{A}}_{M}-\bar{O}\right)=0,\\ \left(P_{j}-C-T_{j}\left(P_{1}-B_{M}\right)\right)\left({\bar{P}}_{j}-\bar{C}-{\bar{T}}_{j}\left({\bar{P}}_{1}-{\bar{B}}_{M}\right)\right)-\left(B_{M}-C\right)\left({\bar{B}}_{M}-\bar{C}\right)=0,\\ j=2,\ldots ,m. \end{array}$$

For *n* FTPs, we have

(21) $$\begin{array}{cc} \left(S_{k}\left(B_{F}-C\right)-O+C-Q_{k}\left(A_{F}-O\right)\right)\left({\bar{S}}_{k}\left({\bar{B}}_{F}-\bar{C}\right)-\bar{O}+\bar{C}-{\bar{Q}}_{k}\left({\bar{A}}_{F}-\bar{O}\right)\right) & \\ -\left(B_{F}-A_{F}\right)\left({\bar{B}}_{F}-{\bar{A}}_{F}\right)=0.\;k=2,\ldots ,n. & \end{array}$$

To decrease the number of unknowns, we establish the relationship between $A_{M}$, $B_{M}$ and $A_{F}$, $B_{F}$. In a complex plane, a unit vector can represent rotation operation; therefore, the rotation from the *x*-axis of the fixed frame to vectors $A_{M}-O$ and $B_{M}-C$ can be denoted, respectively, as

(22) $$\begin{array}{c} Q_{M}=\frac{A_{M}-O}{\sqrt{\left(A_{M}-O\right)\left({\bar{A}}_{M}-\bar{O}\right)}},\;S_{M}=\frac{B_{M}-C}{\sqrt{\left(B_{M}-C\right)\left({\bar{B}}_{M}-\bar{C}\right)}}. \end{array}$$

The rotation operations of input link OA and output link BC from the first MTP to first FTP can be denoted, respectively, as

(23) $$\begin{array}{c} Q_{MF}=Q_{M}e^{-i\phi_{1}},\;S_{MF}=S_{M}e^{-i\psi_{1}}. \end{array}$$

Now, the relationship between $A_{M}$, $B_{M}$ and $A_{F}$, $B_{F}$, and its conjugates are obtained,

(24) $$\begin{array}{cc} & A_{F}=Q_{MF}\left(A_{M}-O\right)+O,\;B_{F}=S_{MF}\left(B_{M}-C\right)+C,\\ & {\bar{A}}_{F}={\bar{Q}}_{MF}\left({\bar{A}}_{M}-\bar{O}\right)+\bar{O},\;{\bar{B}}_{F}={\bar{S}}_{MF}\left({\bar{B}}_{M}-\bar{C}\right)+\bar{C}. \end{array}$$

Substituting Equation (24) into Equation (21) to eliminate $A_{F}$, $B_{F}$ and ${\bar{A}}_{F}$, ${\bar{A}}_{F}$ and obtain

(25) $$\begin{array}{c} \left(S_{k}S_{MF}\left(B_{M}-C\right)-O+C-Q_{k}Q_{MF}\left(A_{M}-O\right)\right)({\bar{S}}_{k}{\bar{S}}_{MF}\left({\bar{B}}_{M}-\bar{C}\right)-\bar{O}+\bar{C}\\ -{\bar{Q}}_{k}{\bar{Q}}_{MF}\left({\bar{A}}_{M}-\bar{O}\right))-\left(S_{MF}\left(B_{M}-C\right)+C-Q_{MF}\left(A_{M}-O\right)-O\right)({\bar{S}}_{MF}({\bar{B}}_{M}\\ -\bar{C})+\bar{C}-{\bar{Q}}_{MF}\left({\bar{A}}_{M}-\bar{O}\right)-\bar{O})=0.\;k=2,\ldots ,n. \end{array}$$

The constraint equations for independent combination for mixed generation are combination of Equations (20) and (25). There are eight unknowns, O, C, $\bar{O}$, $\bar{C}$, $A_{M}$, $B_{M}$, ${\bar{A}}_{M}$ and ${\bar{B}}_{M}$. The feasible independent combinations of MTPs and FTPs are {t=0,m=4,n=3} and {t=0,m=3,n=5}.

## 3. Numerical Examples

In this section, we present numerical examples to illustrate the mixed constraint equations and determine non-defective four-bar linkages. For lack of space, we do not show numerical examples for all cases of mixed constraint equations. According to the number of unknowns in the nonlinear equations, we select one for maximum number of unknowns, the combination of {t=1,m=3,n=5}, and one for minimum number of unknowns, the combination of {t=3,m=3,n=3}, respectively.

### 3.1. Example for {t=1,m=3,n=5}

For combination of {t=1,m=3,n=5}, the four-bar linkage achieves 3 MTPs and 5 FTPs with a CTP. We specified the values and order of these positions in Table 2.

In this example, the CTP is the fourth task position that the four-bar linkage passes through. substituting these values into Equations (15) and (16) to form a polynomial system with eight equations and eight unknowns. In order to obtain all isolated solution sets, Bertine [21,26] is applied to solve the polynomial system. To decrease the number of tracking path, here, we give a group division $\left\{\left\{O,C,A,B\right\},\left\{\bar{O},\bar{C},\bar{A},\bar{B}\right\}\right\}$, and the multi-homogenious Bezout number [23,27] is 70. After 70 initial values are tracked, 60 solution sets are obtained. After checking the conjugates of O,C,A,B and $\bar{O},\bar{C},\bar{A},\bar{B}$, 12 solution sets were remained. Then, filtering those which are defective linkages, a solution set is obtained which are

(26) $$\begin{array}{ccc} & & O=131.08+65.34i,\;C=163.97+70.08i,\\ & & A=118.64+81.17i,\;B=157.88+90.23i. \end{array}$$

**Figure 4:**
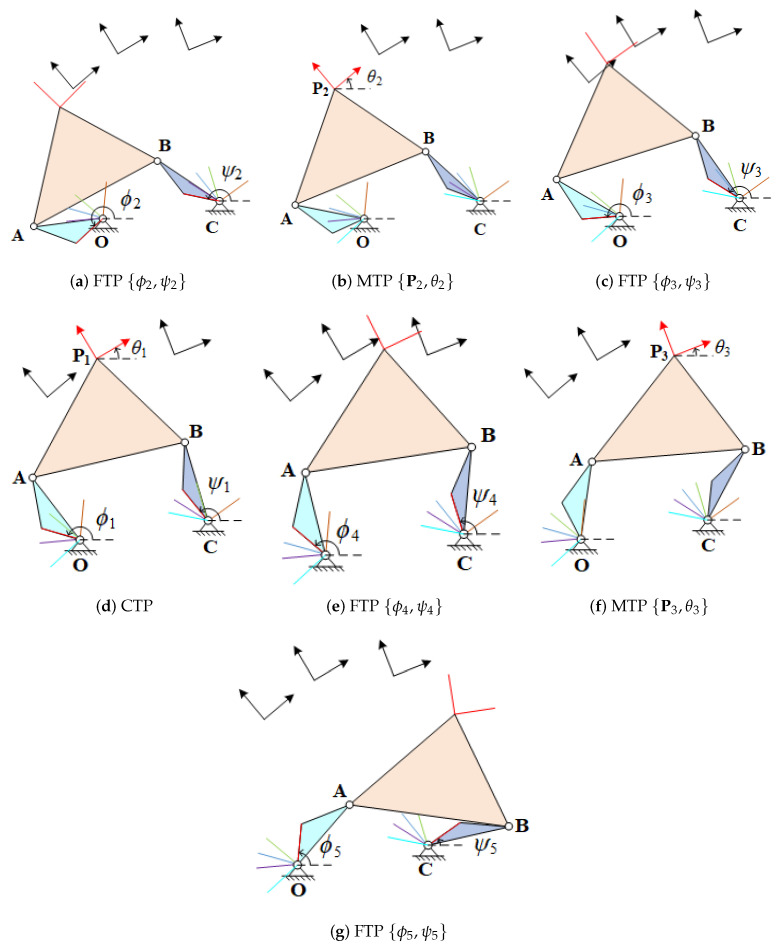
The four-bar linkage moves through each of prescribed task positions of {t=1,m=3,n=5} smoothly.

The images of the four-bar linkage moves through each of prescribed task positions of {t=1,m=3,n=5} smoothly are displayed in Figure 4:

### 3.2. Example for {t=3,m=3,n=3}

For combination of {t=3,m=3,n=3}, the four-bar linkage achieve 3 MTPs and 3 FTPs, and each of MTPs and FTPs are passed through simultaneously. We specified the values and order of these task positions in Table 3.

Substituting the values into Equation (19) can obtain a linear system which includes four equations and four unknowns. The solution set is

(27) $$\begin{array}{ccc} & & O=29.04+14.81i,\;C=32.94+4.99i,\\ & & A=21.65+22.74i,\;B=45.04+28.41i. \end{array}$$

**Figure 5:**
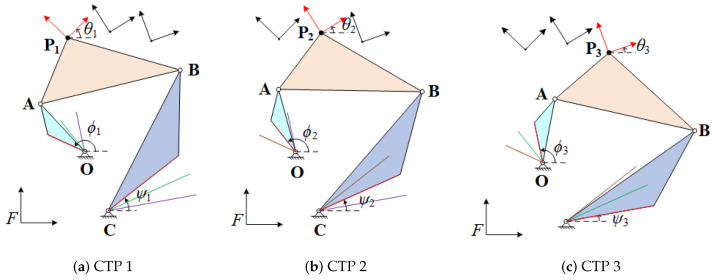
The four-bar linkage moves through each of the prescribed task positions of {t=3,m=3, n=3} smoothly.

The images of the four-bar linkage move through each of prescribed task positions of {t=3,m=3,n=3} smoothly are displayed in Figure 5.

## 4. Conclusions

This paper presents a numerical procedure to synthesize four-bar linkage for mixed motion generation and function generation. The synthesis procedure is divided into two categories, dependent combination and independent combination. The feasible combinations are {t=1,m=4,n=3}, {t=1,m=3,n=5}, {t=2,m=4,n=2}, {t=2,m=3,n=4}, {t=3,m=3,n=3} for dependent combination, and {t=0,m=4,n=3}, {t=0,m=3,n=5} for independent combination. Fully constrained vector loop equations for each of the feasible combinations are formulated and analyzed in a complex plane based on separate synthesis of motion and function generation. Numerical examples are presented to demonstrate the mixed synthesis procedure and determine defect-free four-bar linkages.

## Figures and Tables

**Table 1:** Feasible dependent combinations of MTPs and FTPs.

t=1	m=4	n=3
	m=3	n=5
t=2	m=4	n=2
	m=3	n=4
t=3	m=3	n=3

**Table 2:** The prescribed MTPs and FTPs for {t=1,m=3,n=5}.

Order	MTPs	FTPs
1		$\left\{\phi_{2},\psi_{2}\right\}=\left\{223.50^{\circ },167.73^{\circ }\right\}$
2	$\left\{P_{2},\theta_{2}\right\}=\left\{122.75+101.95i,39.77^{\circ }\right\}$	
3		$\left\{\phi_{3},\psi_{3}\right\}=\left\{186.57^{\circ },146.65^{\circ }\right\}$
4	$\left\{P_{1},\theta_{1}\right\}=\left\{135.34+111.79i,30.34^{\circ }\right\}$	$\left\{\phi_{1},\psi_{1}\right\}=\left\{164.20^{\circ },128.68^{\circ }\right\}$
5		$\left\{\phi_{4},\psi_{4}\right\}=\left\{139.87^{\circ },106.15^{\circ }\right\}$
6	$\left\{P_{3},\theta_{3}\right\}=\left\{155.55+112.96i,21.70^{\circ }\right\}$	
7		$\left\{\phi_{5},\psi_{5}\right\}=\left\{84.02^{\circ },35.13^{\circ }\right\}$

**Table 3:** The prescribed MTPs and FTPs for {t=3,m=3,n=3}.

Order	MTPs	FTPs
1	$\left\{P_{1},\theta_{1}\right\}=\left\{26.22+33.73i,46.16^{\circ }\right\}$	$\left\{\phi_{1},\psi_{1}\right\}=\left\{155.98^{\circ },37.54^{\circ }\right\}$
2	$\left\{P_{2},\theta_{2}\right\}=\left\{33.29+34.75i,31.76^{\circ }\right\}$	$\left\{\phi_{2},\psi_{2}\right\}=\left\{128.59^{\circ },24.00^{\circ }\right\}$
3	$\left\{P_{3},\theta_{3}\right\}=\left\{40.07+33.25i,19.68^{\circ }\right\}$	$\left\{\phi_{3},\psi_{3}\right\}=\left\{102.14^{\circ },9.84^{\circ }\right\}$

## Data Availability

No data available.
